# SGLT2 inhibitor versus carbohydrate-restricted isocaloric diet: reprogramming substrate oxidation in type 2 diabetes

**DOI:** 10.1186/s13098-023-00990-6

**Published:** 2023-02-19

**Authors:** Hiroyuki Igarashi, Hiroshi Uchino, Momoko Kanaguchi, Kaori Hisanaga, Genki Sato, Fukumi Yoshikawa, Masashi Furuta, Naohiro Washizawa, Shuki Usui, Masahiko Miyagi, Takahisa Hirose

**Affiliations:** 1 Division of Diabetes, Metabolism and Endocrinology, Department of Internal Medicine, Toho University Graduate School of Medicine, 6-11-1 Omori-Nishi, Ota-Ku, Tokyo, 143-8541 Japan; 2 Nutrition Therapy Center, Toho University Omori Medical Center, Tokyo, 143-8541 Japan; 3 Division of Diabetes and Endocrinology, Department of Internal Medicine, Saiseikai Kanagawaken Hospital, Yokohama, Kanagawa Japan

**Keywords:** Sodium/glucose cotransporter-2 inhibitor, Carbohydrate-restricted isocaloric diet

## Abstract

**Objective:**

Based on the whole-body energy metabolism and insulin action, the difference between increased excretion of carbohydrate in urine by SGLT2i and reduced same amount of oral carbohydrate intake are scarce. This study aimed to compare the effect of carbohydrate availability with reduced oral intake (carbohydrate-restricted isocaloric diet: CRIC diet) or lost in urine, as urinary glucosuria on sodium/glucose cotransporter-2 inhibitor (SGLT2i) treatment, focus on the insulin requirement and the macronutrient oxidation within insulin treated type 2 diabetes.

**Methods:**

This is randomized 3-arm open-label prospective study. Subjects treated with titrated basal-bolus insulin regimen subsequent to three diet regimens, control diet (CON), administration of canagliflozin 100 mg/day to CON (SGLT2i), or CRIC diet, with a week admission to the endocrinology ward followed by 12 weeks outpatients’ management. The main outcome measures including the total insulin dose (TID) required to achieve euglycemia, fasting and postprandial energy expenditure (EE) and respiratory quotient (RQ) at 1-week and 12-week.

**Results:**

We enrolled 23 patients with type 2 diabetes (male/female: 14/9, age: 53.6 ± 14.2 years, body mass index: 26.9 ± 4.8 kg/m^2^, HbA1c: 12.5 ± 1.6%). The TID was similar with CON and SGLT2i at both 1 and 12-weeks. Although comparable net carbohydrate availability in SGLT2i and CRIC groups, the TID was significantly higher in the CRIC (p = 0.02) compare to the SGLT2i at both 1 and 12-weeks. Fasting EE was similar in all groups, postprandial EE was significantly elevated in the SGLT2i and CRIC groups compared to the CON group (p = 0.03 and 0.04). Compare to the CON, lower basal fasting RQ (p = 0.049) and decreased delta-RQ (postprandial RQ/fasting RQ) indicated continuous lipid substrate utilization in the SGLT2i (p = 0.04) and CRIC (p = 0.03) groups.

**Conclusion:**

The CRIC diet resulted in a similar fasting and postprandial EE and substrate oxidation compared to the SGLT2i. The increased insulin requirement in the CRIC diet indicates that a relatively highly lipid and protein consumption, compared to the SGLT2i and CON, may influence insulin requirement.

**Supplementary Information:**

The online version contains supplementary material available at 10.1186/s13098-023-00990-6.

## Introduction

The dietary composition affects glycemia and insulin sensitivity in patients with type 2 diabetes; however, a diet composed of only specific macronutrients may be less beneficial and difficult to tolerate for a long period [1, 2]. Pharmacotherapy, including renal sodium/glucose cotransporter-2 inhibitors (SGLT2i), that can improve glycemic control when combined with insulin therapy could mitigate metabolic stress due to nutritional intake and confer additional benefits on health outcomes in type 2 diabetes [3]. The inhibition of SGLT2 in the proximal convoluted tubule suppresses sodium and glucose reabsorption, promotes their elimination in urine, improves glycemic control, and ultimately ameliorates the progression of diabetes-related complications [4, 5]. SGLT2i treatment in humans decreases body weight [6], increases the reliance on fat as an energy source [3], and improves cardiac function, blood pressure, and cardiovascular outcomes [7].

Although SGLT2i eliminates a certain amount of glucose in urine, resulting whole-body net calorie loss, it is unclear whether the treatment-associated benefit contributes to energy loss [4, 5]. Therefore, we reduced the carbohydrate intake, which corresponded to the amount of urinary glucosuria resulting after SGLT2i treatment, assessing whether the effects of SGLT2i were related to hypoenergetic conditions. We formulated a carbohydrate restricted isocaloric (CRIC) diet to address these shortcomings, which eliminates the amount of carbohydrate equivalent to that of glucose (approximately 50 g/day^3^) dissipated in the urine by SGLT2i, allowing us to evaluate systemic macronutrient metabolisms and energy expenditure (EE) in type 2 diabetes.

Type 2 diabetes is predominantly characterized by insulin resistance, its progression is associated with insulin deficiency requiring insulin administration [8]. Previous studies have failed to illustrate an essential aspect, i.e., if SGLT2i pharmacotherapies for type 2 diabetes have a permissive or additive effect on the dose of insulin [9] and if they exert any impact on the methods used to achieve carbohydrate restrictions.

Thus, we examined the insulin doses required to achieve euglycemia in insulin-treated type 2 diabetes patients with SGLT2 inhibitors and the CRIC diet. In a previous study, we achieved euglycemia by eliminating glucose in urine by adding SGLT2i and reduced the time required to achieve euglycemia for the management of insulin-treated type 2 diabetes [3].

Furthermore, the impact of nutritional carbohydrate energy availability (i.e., the result of excreting carbohydrate via urine) on the EE [10] and respiratory quotient (RQ) [11] was investigated in this study. We also described the effect of these modalities on macronutrient oxidation, i.e., the capacity to switch to fuel oxidation to adjust for fuel availability (i.e., metabolic flexibility), and the selective substrate energy utilization using indirect calorimetry in the fasting and postprandial states.

## Materials and methods

### Study design and ethics approval

This 12-week long open-label, randomized (1:1:1), prospective trial enrolled inpatients who were hospitalized (recruited from the outpatient department) at the Faculty of Medicine, Toho University School of Medicine, Tokyo, Japan, to evaluate the effects of the co-administration of an SGLT2 inhibitor with the CON diet or CRIC diet with multiple daily insulin treatments. This study was conducted between November 2016 and January 2019. The protocol was reviewed by the Japanese authorities in accordance with local regulations followed by review and approval by the institutional review board of Toho University Omori Medical Center (M1618917, 11717238). The study was conducted in accordance with the International Conference on Harmonization Guidelines for Good Clinical Practice and the Declaration of Helsinki [12]. The study was registered with the National University Hospital Medical Information Network (UMIN Clinical Trials Registry: UMIN0000 27262).

### Participants

Patients with type 2 diabetes requiring hospitalization to control hyperglycemia were eligible for enrollment in this study if they were aged above 20 years, had hemoglobin A1c (HbA1c) more than 10% and a daily mean pre-prandial blood glucose concentration of more than 11 mmol/L (200 mg/dL). Patients who had been administered any antidiabetic medication previously (including insulin) were excluded. The exclusion criteria were as follows: type 1 diabetes or secondary diabetes caused by other underlying conditions, myocardial infarction < 3 months before enrollment or known heart failure, history of hypersensitivity to the study drugs, history diabetic coma, those at risk of diabetic coma, severe liver disease, severe renal disease, severe pancreatic disease, hemoglobin (Hb) < 11 g/dL, current malignancy, platelet count < 100,000/mm^3^, severe diabetic neuropathy, proliferative retinopathy, serious infection, recent surgery or severe trauma, or excessive alcohol consumption. Pregnant women or possibly pregnant women were also excluded. Patients judged unsuitable for the study by the attending physician were also excluded. Written informed consent was obtained from all participants.

### Interventions

Twenty-three patients with type 2 diabetes were treated on the basis of the same basal-bolus insulin titration algorithm. Patients received meals thrice a day (at 08.00, 12.00, 18.00 h) in an inpatient setting for a week followed by outpatient settings for total 12 weeks study period. They were randomized to the control diet (CON) group, administration of canagliflozin 100 mg/day to the control diet (SGLT2) group, and CRIC diet groups. The control diet was designed to provide 28 kcal/kg multiple by ideal body weight with ~ 60% of total daily energy requirements from carbohydrates, ~ 25% from fat, and ~ 15% from protein. The carbohydrate-restrictive iso-caloric diet group (CRIC) intervention consisted of ~ 54% of energy from carbohydrate, ~ 29% of energy from fat, 17% of energy from protein with energy contents of 25 kcal·kg-1 ideal body weight·day-1. In control group (CON) and SGLT2i groups, control diet was provided and CRIC diet for CRIC group, by registered dietitians with monthly medical dietary advice to receive allocated intervention for three months. Participants received education in medical diet counselling in addition to the standard outpatient nutrition education as described for the control group. The medical diet counselling consists of four sessions of an hour each, two sessions in inpatient and followed by two sessions in outpatient clinic. The counselling uses trained dietitians following a planned curriculum which include experience-based learning with problem-solving exercises, hands-on activities, short theoretical presentations, discussions of motivational aspects and coping strategies. The counselling integrates peer modelling, skills development, goal setting, observational learning and social support into the counselling content and activities. The training includes identifying carbohydrates in food, reading carbohydrate tables, calculating the carbohydrate content from food labels, tables and food guide text, and use of a personalized carbohydrate plan with guiding suggestions for daily intake of carbohydrates at meals based on personal dietary recordings including plasma glucose measurements. All groups received the same insulin regimen. Randomization was performed using the minimization method with the following background factors: blood glucose level and age. The target fasting and postprandial blood glucose concentrations were 4.5–6.1 mmol/L (80–110 mg/dL) and 4.5–7.8 mmol/L (80–140 mg/dL), respectively. The insulin dose was adjusted using an insulin algorithm adopted from a previous study [3], according to the blood glucose concentration measured by the previous day using a glucose meter (One Touch; Johnson & Johnson). The starting dose of insulin was 0.2 IU/kg/day (1:1 basal–bolus). The basal insulin dose (insulin glargine) was increased by 2, 3 and 4 IU for fasting blood glucose concentrations elevations of 6.1–7.7 mmol/L (110–139 mg/dL), 7.8–9.9 mmol/L (140–179 mg/dL) and more than 10 mmol/L (> 180 mg/dL), respectively. The bolus insulin dose (insulin aspart) was increased by 2 and 3 IU for postprandial blood glucose concentrations of 7.8–9.9 mmol/L (140–179 mg/dL) and more than 10 mmol/L (> 180 mg/dL), respectively. The schematic description that represent the outline in this study (Additional file 1).

### Procedures and study design

Patients received meals thrice a day with insulin treatment alogism in an inpatient setting for a week and followed every month to 12 weeks study period. After an overnight fast and a 1-h basal period, patients consumed a mixed meal (within 10 min) comprising 60% carbohydrate, 25% fat, and 15% protein with a total calorie content of 28 kcal/kg for the CON. The SGLT2 group provided the CON diet with adding canagliflozin 100 mg/day. The CRIC diet regimen (n = 7) was as follows: 25 kcal/kg ideal body weight; 54% of total caloric intake was based on carbohydrate based on 28 kcal/kg SBW with a decreased dose of approximately 50 g. The feasibility of the diets tested in this study was confirmed by registered nutritionists, who contacted patients during treatment on a monthly basis. All registered nutritionists independently evaluated the patients’ dietary records [13, 14]. Indirect calorimetry (METALYZER® 3 B; CORTEX, Germany) was conducted the same environment on the day before the initiation of treatment and 1- and 12-weeks post-treatment. The measurements were performed for 10-min periods at the following times: − 30 to − 20 min before the meal, and 30–60, 90–120, 150–180, and 210–240 min after the meal. Blood samples were collected at the same time points.

### Efficacy end-points

The primary efficacy end-point was the total daily dose of insulin required to achieve euglycemia, which was defined as a daily mean pre-prandial blood glucose concentration of ≤ 7.8 mmol/L (140 mg/dL). The secondary efficacy end-points including EE and RQ, as measured by indirect calorimetry performed one day before treatment day, day 7 and after 12 weeks. Oxygen consumption and carbon dioxide production were measured to calculate the EE and RQ. According to the Weir equation, EE was calculated without using urinary urea nitrogen levels [14]. Protein oxidation was determined from 24-h urinary nitrogen excretion, and the carbohydrate and lipid oxidation rates were determined from the non-protein RQ. EE and RQ were assessed at the steady state, which was defined as the 10-min time period during which the average minute-by-minute changes in oxygen consumption and carbon dioxide production were < 10% and the average RQ change was < 5% [16]. The average RQ and EE at the steady state were measured at each 10-min recording period.

The exploratory end-points included the changes in daily urinary glucose excretion, 3β-hydroxybutyrate, acetoacetate concentrations, body weight, systolic blood pressure, and diastolic blood pressure.

### Drug safety

The safety parameters were assessed in terms of adverse events, hypoglycemic events. and laboratory test values. The adverse events were evaluated by the attending physicians. The absence of diabetic ketoacidosis was evaluated by measuring plasma and urine ketone bodies.

### Body composition

Body composition was assessed pre-and post-intervention after 12 weeks using the InBody Composition Analyzer (InBody Japan, Tokyo, Japan).

### Statistical analysis

The mechanism and the magnitude of the SGLT2i by which improves hyperglycemia were mainly reported in patients with oral antidiabetic treatment [17, 18]. To our knowledge, only few studies were elucidated to evaluate the insulin requirement with SGLT2i. To address this criticism, the sample size calculation in this study was based on the insulin requirement to achieve euglycemia in our previous study [3]. Regarding to our previous study, the dose of daily total insulin requirement difference between two groups, multiple insulin injection alone and adding SGLT2 inhibitor to multiple insulin injection, was 0.14U/kg/day and the standard deviation was 0.16 U/kg/day. This allows us to calculate the number in this study was 24 participants in three arms. Twenty-four patients were required (8 per group) to achieve a significance level of 5% and statistical power of 80%. Therefore, we planned to enroll 10 patients per group after accounting for potential dropouts. The efficacy data were analyzed in the full analysis set, defined as all patients who were registered in the study, randomized to either group, who received at least one dose of the study drug, and in whom some data were recorded after the start of treatment. Safety analyses were performed in all registered patients and those who received at least one dose of the allocated study treatment. The results were presented as the mean ± standard deviation or median (interquartile range) for normally and non-normally distributed variables, respectively. The within-group changes in variables were analyzed using the paired *t*-test and Wilcoxon signed-rank test for normally and non-normally distributed variables, respectively. Two-way ANOVA for time-series data. Statistical significance was set at p-values < 0.05. All analyses were conducted using JMP version 12 (www.JMP.com).

## Results

A total of 26 patients were initially screened for this study upon admission. However, three patients withdrew consent for participation, and the remaining 23 were randomly assigned to the CON, insulin + canagliflozin (SGLT2i) and CRIC (Table 1) groups. The baseline characteristics of the patients in three groups were comparable (Table 1). The mean ± standard deviation of the duration of diabetes (0.8 ± 0.6, 4.4 ± 2.6, 4.5 ± 2.1 years), age (50.4 ± 14.0, 53.8 ± 15.2, 56.9 ± 15.6 years), sex (male/female; 4/4, 6/2, 4/3), height (cm; 165.5 ± 12.4, 165.2 ± 9.5, 160.9 ± 7.8), weight (75.9 ± 16.6, 672.7 ± 18.7, 65.4 ± 9.4 kg), BMI (27.7 ± 5.1, 26.3 ± 5.1, 25.2 ± 3.0 kg/m^2^), total body muscle mass (49.2 ± 12.3, 51.1 ± 13.1, 42.8 ± 6.4 kg) and total body fat mass (23.5 ± 12.0, 18.4 ± 6.5, 19.9 ± 5.5 kg) were similar in the CON, SGLT2i and CRIC groups, respectively. After the 12-weeks treatment period, weight, BMI, total body fat mass changes were similar in all three groups (Table 1). The post 12-weeks percentages changes in total body muscle mass in the CRIC significantly increased compared to the CON and the SGLT2i (Table 1).

**Table 1:** Characteristics and anthropometric measurements of the study subjects

	Pre (0w)			Post treatment (12w)					
	Control	SGLT2i	CRIC	Control	%△Control	SGLT2i	%△SGLT2i	CRIC	%△CRIC
Duration of diabetes (years)	0.8 ± 0.6	4.4 ± 2.6	4.5 ± 2.1	–	–	–	–	–	–
Age (years)	50.4 ± 14.0	53.8 ± 15.2	56.9 ± 15.6	–	–	–	–	–	–
Sex (Male/Female)	4/4	6/2	4/3	–	–	–	–	–	–
Height (cm)	165.5 ± 12.4	165.2 ± 9.5	160.9 ± 7.8	–	–	–	–	–	–
Weight (kg)	75.9 ± 16.6	72.7 ± 18.7	65.4 ± 9.4	71.7 ± 15.8	-2.2 ± 6.8	70.2 ± 16.5	1.1 ± 5.0	66.8 ± 9.8	2.4 ± 6.2
BMI (kg/m^2^)	27.7 ± 5.1	26.3 ± 5.1	25.2 ± 3.0	26.2 ± 4.1	-2.2 ± 6.8	25.9 ± 4.4	1.1 ± 5.0	25.8 ± 2.8	2.4 ± 6.2
Total body muscle mass (kg)	49.2 ± 12.3	51.1 ± 13.1	42.8 ± 6.4	50.5 ± 15.6	2.7 ± 6.2	50.3 ± 12.7	3.0 ± 2.0	46.6 ± 8.1	8.6 ± 5.8 * **
Total body fat mass (kg)	23.5 ± 12.0	18.4 ± 6.5	19.9 ± 5.5	17.9 ± 10.1	-15.5 ± 25.2	16.8 ± 4.7	-2.1 ± 20.3	17.3 ± 6.3	-12.6 ± 16.7

Data are mean ± standard deviation

%△Control, %△SGLT2i, %△CRIC: percentage differs from 12 to 0w in various parameters

SGLT2i, renal sodium/glucose cotransporter-2 inhibitor; CRIC, carbohydrate restricted iso-caloric diet

*p < 0.05 (t test) %△Control vs %△CRIC

**p < 0.05 (t test) %△SGLT2i vs %△CRIC

### Efficacy end-point: insulin requirement

**Fig. 1:**
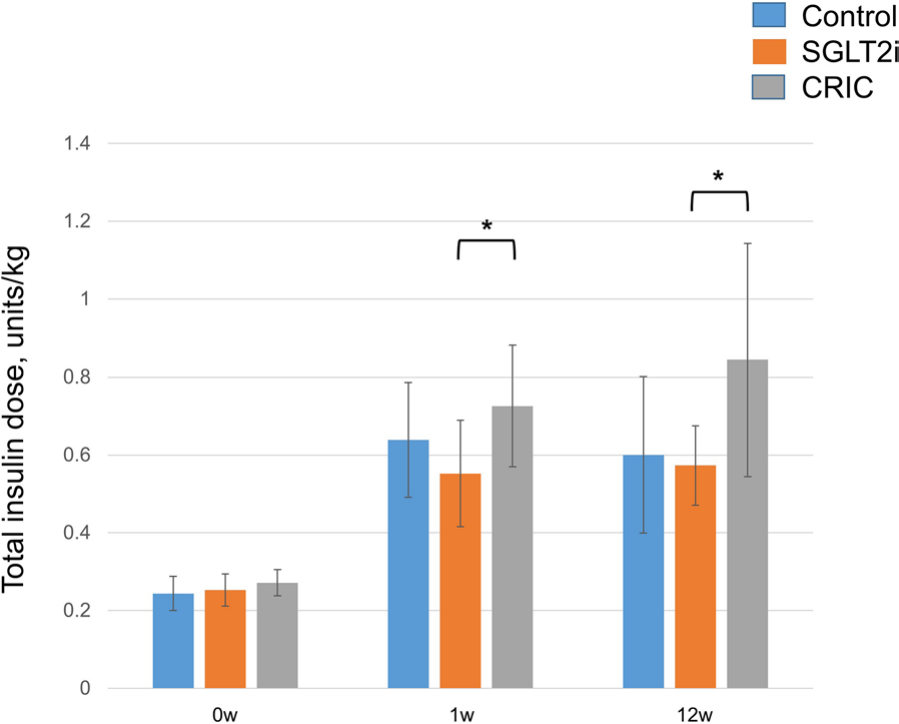
Total insulin dose to achieve euglycemia at baseline, 1 week and 12 weeks. Blue bars represent CON. Orange bars represent SGLTi. Gray bars represent CRIC. Data are mean ± SD. FFA, free fatty acid; CRIC, carbohydrate restricted-iso-caloric diet

**Fig. 2:**
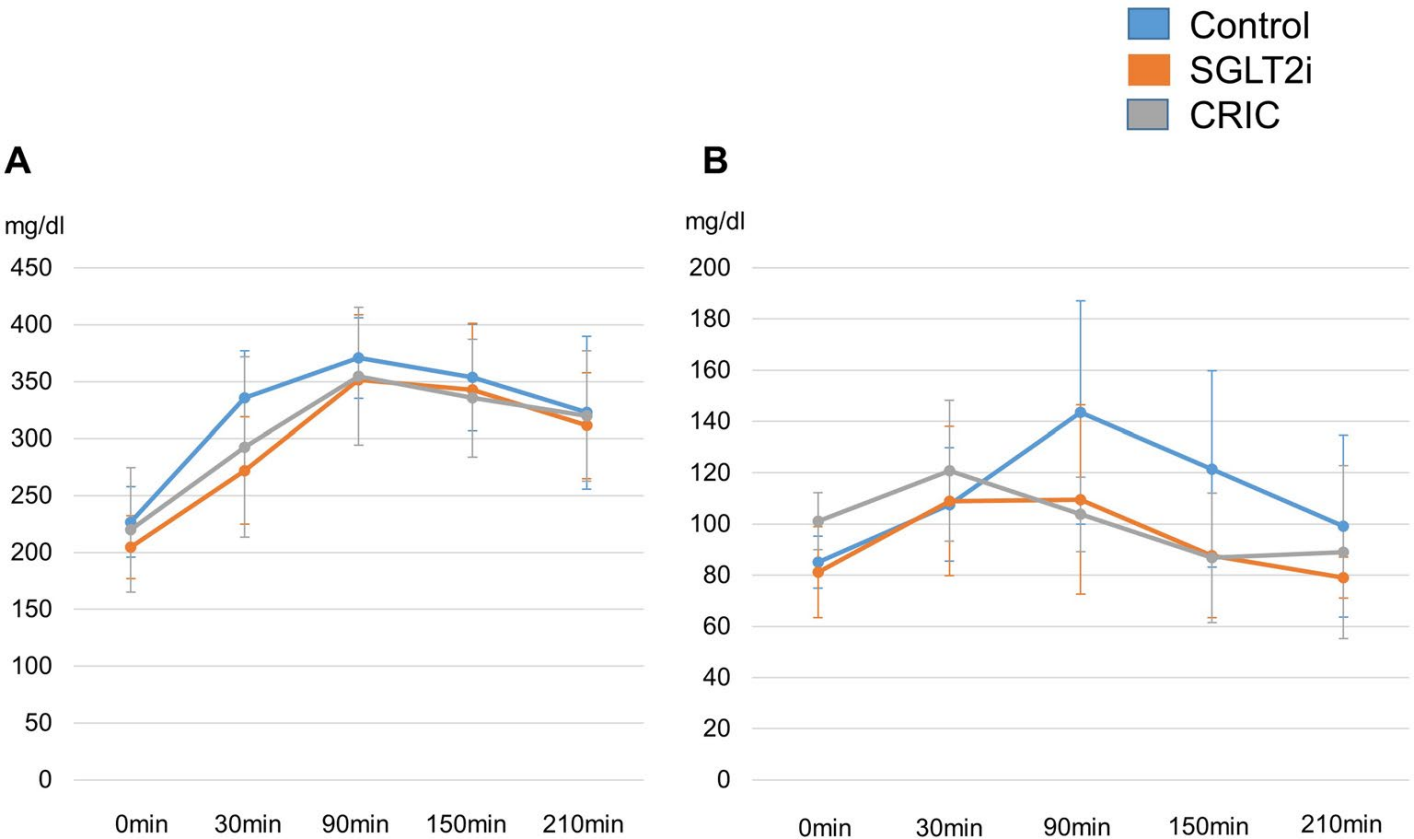
Post-prandial plasma glucose concentrations at baseline (**A**) and 12 weeks (**B**). Blue line represent CON. Orange line represent SGLTi. Gray line represent CRIC. Data are mean ± SD. FFA, free fatty acid; CRIC, carbohydrate restricted-iso-caloric diet

The co-administration of canagliflozin was associated with significantly lower total insulin doses compared to the CRIC diet during post-treatment weeks 1 and 12 (0.55 ± 0.14 vs 0.73 ± 0.16 U/kg and 0.57 ± 0.10 vs 0.84 ± 0.30 U/kg, p < 0.05; Fig. 1). The total insulin doses were similar between the CON and SGLT2i groups. The post-prandial glycemic response was similar in all three groups at pre- and 12 weeks (Fig. 2A and B). The area under the curve of the mean pre-prandial blood glucose obtained at baseline was comparable for all groups (data not shown). The level of urinary glucose excretion was higher in all groups at baseline, as might be expected considering the severity of hyperglycemia. Urinary glucose excretion declined 12 weeks post-treatment in the CON and CRIC groups after the initiation of insulin therapy. However, it remained higher in the SGLT2i group (data not shown). The serum 3β-hydroxybutyrate and acetoacetate concentrations tended to decrease in all three groups without significant differences.

### Lipids

**Fig. 3:**
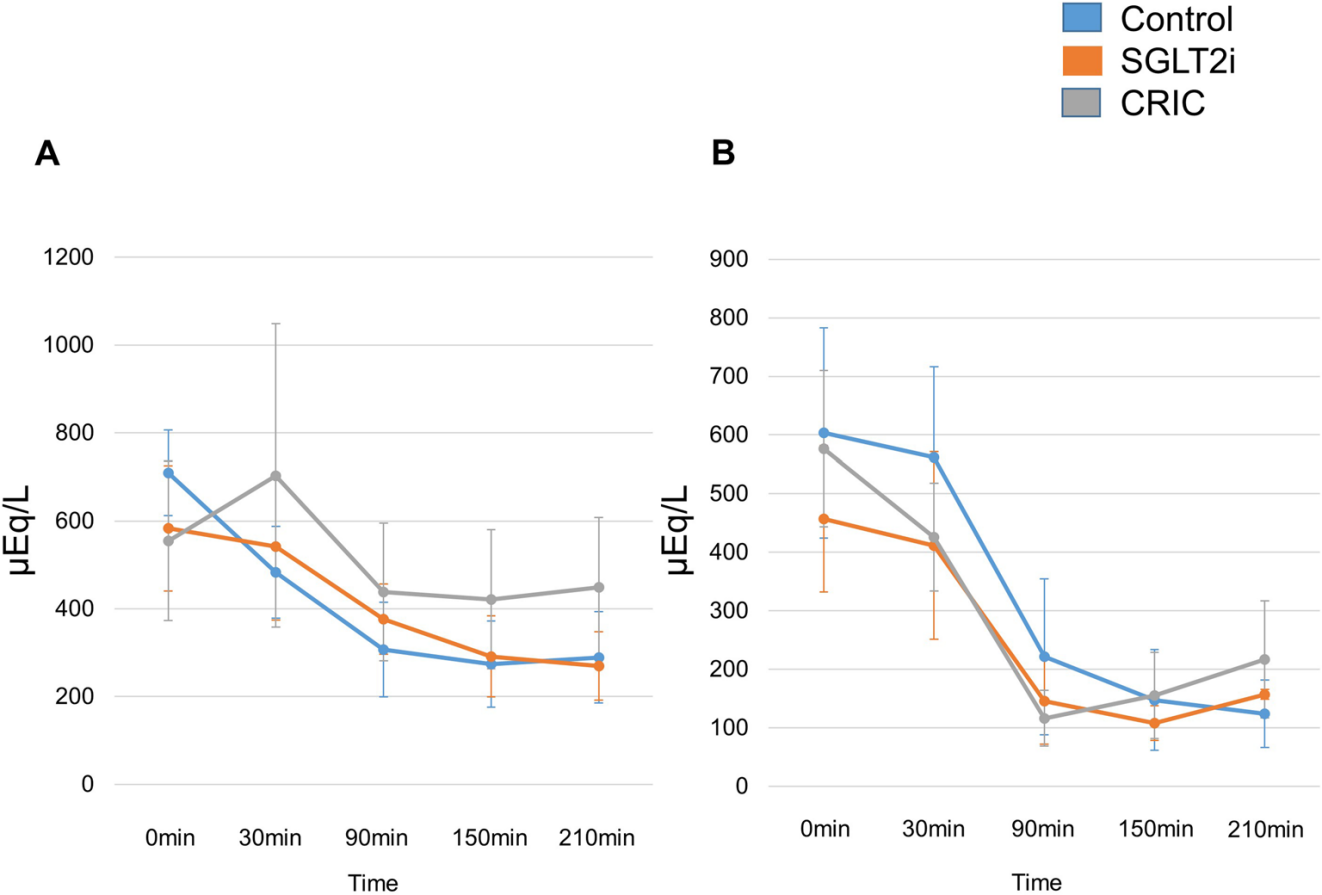
Post-prandial plasma FFA levels at baseline (**A**) and 12 weeks (**B**). Blue line represent CON. Orange line represent SGLTi. Gray line represent CRIC. Data are mean ± SD. FFA, free fatty acid; CRIC, carbohydrate restricted-iso-caloric diet

In Table 2, there were no significant changes in the HbA1c, HDL cholesterol, TG, serum leptin, and high molecular-weight adiponectin at baseline in all groups. The total cholesterol and LDL were higher at baseline in the CRIC group compared to the CON and SGLT2i groups (Table 2). After the 12w post -treatment, these variables were consistently higher in the CRIC group compared to the CON group. The %ΔLDL cholesterol (12w post-treatment-0w pre-treatment/0w pre-treatment) was significantly attenuated in the CRIC group compared to the SGLT2i group at 12 weeks. The total cholesterol, HDL and LDL were significantly lower in CRIC compared to the CON at 12w post-treatment period. The serum total ketone body concentration before the initiation of treatment was 557.6 ± 753.1, 329.2 ± 314.4, 751.7 ± 1172.0 μmol/L in the CON, SGLT2i and CRIC groups, respectively. Regarding the pre- and post-prandial free fatty acid levels in pretreatment, the specific concentration at 210 min was significantly elevated in CON compared to the SGLT2i (data not shown). However, the overall time-series data represents post-prandial free fatty acid levels were similar in all three groups, in pretreatment and 12 weeks later, according to the two-way ANOVA analysis (Fig. 3A, B).

**Table 2:** Changes of all the laboratory results

	Pre (0w)			Post treatment (12w)					
	Control	SGLT2i	CRIC	Control	%△Control	SGLT2i	%△SGLT2i	CRIC	%△CRIC
HbA1c (%)	12.9 ± 1.8	11.8 ± 1.3	12.8 ± 1.8	5.6 ± 0.6	− 55.5 ± 8.7	6.1 ± 0.6	− 48.4 ± 7.7	6.1 ± 0.7	− 51.9 ± 7.0
T-CHO (mg/dl)	196.4 ± 36.2	192.1 ± 43.7	239.9 ± 29.8**^,^***	164.5 ± 32.8	− 15.5 ± 13.7	199.9 ± 60.5	− 2.0 ± 14.0*	205.0 ± 28.9**	− 14.1 ± 10.8
HDL-C (mg/dl)	44.6 ± 9.1	49.9 ± 13.0	50.9 ± 10.8	47.5 ± 8.1	7.5 ± 10.7	59.4 ± 17.2	18.1 ± 12.7	59.6 ± 10.1**	18.5 ± 15.9
LDL-C (mg/dl)	119.4 ± 28.6	119.3 ± 38.3	163.6 ± 29.2**^,^***	102.4 ± 30.9	− 14.9 ± 19.2	125.4 ± 52.2	− 3.1 ± 14.6	132.7 ± 32.6**	− 18.9 ± 13.8***
TG (mg/dl)	146.3 ± 56.3	166.9 ± 94.8	170.9 ± 119.7	92.4 ± 44.4	− 35.5 ± 25.0	107.0 ± 44.6	− 25.0 ± 25.2	85.6 ± 17.4	− 37.8 ± 22.4
Leptin (ng/ml)	19.4 ± 11.7	12.3 ± 6.7	13.3 ± 6.4	21.3 ± 19.5	11.9 ± 57.0	13.5 ± 8.1	17.6 ± 36.9	22.1 ± 19.0	55.2 ± 87.7
H-M adiponectin (µg/ml)	1.76 ± 0.80	1.81 ± 1.55	1.86 ± 0.66	2.47 ± 1.04	53.7 ± 62.5	3.18 ± 2.34	71.5 ± 44.4	2.85 ± 1.66	50.8 ± 59.3
T-ketone (µmol/l)	557.6 ± 753.1	329.2 ± 314.4	751.7 ± 1172.0	256.8 ± 245.4	− 5.7 ± 87.3	349.0 ± 496.4	− 25.8 ± 71.3	173.0 ± 121.5	− 24.8 ± 91.7
AcAc (µmol/l)	124.1 ± 116.4	96.8 ± 76.8	205.1 ± 298.7	77.8 ± 66.8	4.1 ± 100.6	75.4 ± 76.4	− 28.0 ± 58.5	52.1 ± 40.6	− 29.8 ± 89.3
3-OHBA (µmol/l)	433.4 ± 638.4	232.3 ± 238.5	546.6 ± 873.5	179.0 ± 179.6	− 8.3 ± 84.1	273.6 ± 425.7	− 24.6 ± 78.0	120.9 ± 82.6	− 22.2 ± 93.2

Data are mean ± standard deviation

%△SGLT2i vs %△CRIC. %△Control, %△SGLT2i, %△CRIC: percentage differs from 12 to 0w in various parameters

SGLT2i, renal sodium/glucose cotransporter-2 inhibitor; CRIC, carbohydrate restricted iso-caloric diet; H-M, high molecular; AcAc, acetoacetic acid; 3-OHBA, 3-hydroxybutyric acid

*p < 0.05 (t test) %△Control vs %△SGLT2i

**p < 0.05 (t test) Control vs CRIC

***p < 0.05 (t test) SGLT2i vs CRIC

### Efficacy end-points: whole body energy metabolism

**Fig. 4:**
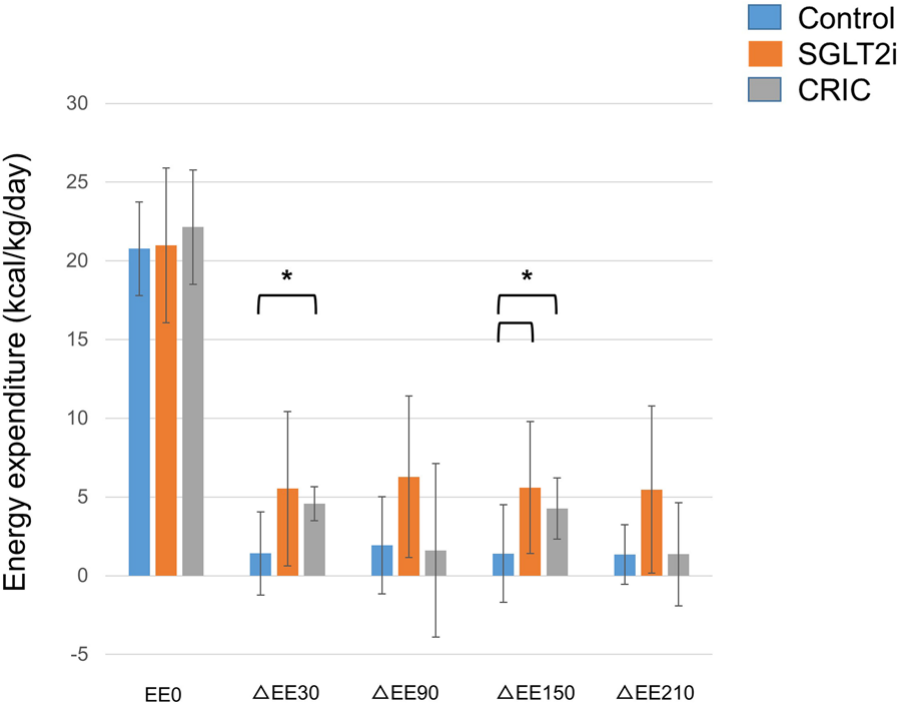
Basal and post-prandial energy expenditure changes. Blue bars represent CON. Orange bars represent SGLTi. Gray bars represent CRIC. Data are mean ± SD. FFA, free fatty acid; CRIC, carbohydrate restricted-iso-caloric diet; EE, energy expenditure. ΔEE represent the difference between fasting EE. *p < 0.05 were considered statistically significant

As shown in Fig. 4, there was no marked difference in the fasting total EE (0) in any group, despite achieving euglycemia with different net calorie balance values (i.e., excessive urinary glucosuria in SGLT2i and low carbohydrate intake in CRIC). The EE measured 30 and 150 min after food intake was higher than that at baseline (EE 0) in the SGLT2i and CRIC groups (p = 0.03 and 0.03, respectively) but not in the CON group, which may be attributed to the thermic effect of nutrient calorie intake. The pre- and post-treatment (12 weeks) protein oxidation did not change in any group.

**Fig. 5:**
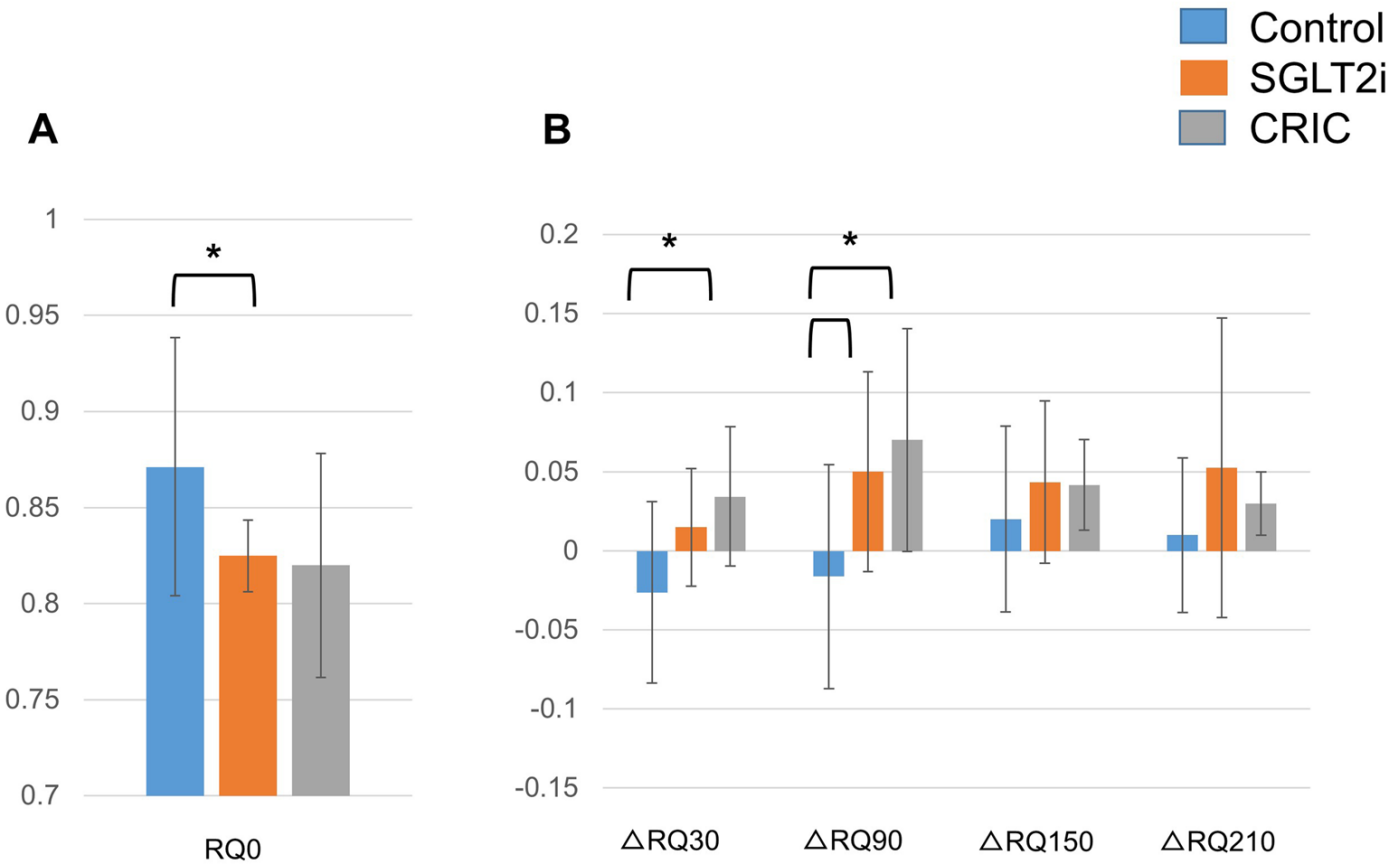
Fasting (**A**) and post-prandial respiratory quotient (RQ) changes (**B**). Blue bars represent CON. Orange bars represent SGLTi. Gray bars represent CRIC. Data are mean ± SD. FFA, free fatty acid; CRIC, carbohydrate restricted-iso-caloric diet; RQ, respiratory quotient. ΔRQ represent the difference between fasting RQ. *p < 0.05 were considered statistically significant

The fasting RQ was marginally lower in the SGLT2i group compared to the CON group (p = 0.049). The delta-RQ (postprandial RQ/fasting RQ) was elevated due to increased carbohydrate substrate utilization at delta-RQ90 (the difference between RQ0min and the RQ90min) in SGLT2i group (p = 0.04) compared to the CON group. In the CRIC group, the delta-RQ30 (the difference between RQ0min and the RQ30min) and delta-RQ90 were significantly increased compared to the CON group (p = 0.03). respectively) (Fig. 5A, B).

### Safety concerns

Treatment-emergent adverse events were not observed in this study population. An independent attending physician determined that none of the symptoms were related to the treatment protocols. None of the patients developed symptoms during the CRIC regimen.

## Discussion

This study aimed to evaluate whether the pathways of carbohydrate availability changes has any effect on insulin requirement and macronutrient substrate utilization with energy expenditure in type 2 diabetes, requiring multiple daily insulin injections. Although reduced net carbohydrate availability in SGLT2i and CRIC groups were similar, the total daily insulin dose required to achieve euglycemia was significantly increased in the CRIC compared to the CON. Fasting EE were similar in all groups and postprandial EE, which related to diet-induced thermogenesis, was significantly elevated in SGLT2i and CRIC groups compared to the CON group. Compare to the CON, lower basal fasting RQ, indicating continuous lipid substrate utilization in SGLT2i and CRIC groups. Increasing early phase post prandial delta-RQ (postprandial RQ/fasting RQ) in SGLT2i and CRIC groups, compare to the CON, suggesting that reduced net carbohydrate availability leads to in part modifying carbohydrate substrate metabolism effectively.

### Insulin requirement

Although, in this prospective 12-week study, the plasma glucose and FFA responses after the meal were comparable in the SGLT2i and CRIC groups (Fig. 3A and B), the total insulin requirement was significantly higher in CRIC compared to SGLT2i during the study period (Fig. 1). Increased total body muscle mass and higher total daily dose of insulin within the CRIC group, implicating increased anabolic demands due to the relatively high proportion of the protein intake [22, 23] Data from series of studies suggest that high protein intake could have detrimental metabolic effects; acute intravenous amino acid infusion or protein ingestion reduces insulin sensitivity [24–27], and habitual high protein intake is associated with insulin resistance and increased risk of developing type 2 diabetes [28–30].

The mechanisms responsible for the adverse effect of the CRIC diet on insulin action are unclear. Whereby, adding an SGLT2 inhibitor to intensive insulin therapy may have some effect in overcoming glucotoxicity by reducing extracellular glucose availability. Studies conducted in cultured myotubes, and isolated skeletal muscles in rodents have demonstrated that amino acids, especially the branched-chain amino acid leucine, can impair insulin-mediated glucose uptake by adenosine monophosphate-activated protein kinase (AMPK)-mediated mammalian target of rapamycin (mTOR) phosphorylation and subsequent negative feedback inhibition of phosphatidylinositol 3-kinase (PI3K)-AKT signaling [31, 32]. Therefore, the discrepancy between the urinary carbohydrate loss and equivalent reduction in dietary carbohydrate intake, consequently to the negative carbohydrate balance in the entire metabolic response in patients with type 2 diabetes, a relatively high protein intake ratio nullifies the beneficial effects of insulin and subsequently increases the daily dose of insulin.

### Impact of the method used to limit carbohydrate availability (low oral intake or high urinary output) on EE

SGLT2 inhibitors enhance urinary glucose excretion, and thus increased whole body energy loss [33]. The resting EE were similar in all groups in this study. We first assumed that SGLT2i group had a lower capacity to maintain negative energy balance (urinary elimination of glucose of approximately 50 g per day accounts for energy loss of 200 kcal/day) during urinary energy loss (i.e., glucosuria), which inhibits adaptive thermogenesis, leading to a suppression in the EE [12, 34]. SGLT2 inhibitors are associated with a reduction in body weight, which appears to be mediated by increased energy loss as a common denominator [20]. The whole-body baseline EE did not differ in the CON, SGLT2i and CRIC groups.

However, energy consumption did not differ between the CON and SGLT2i treatment groups, suggesting that baseline EE was more likely to respond to ingested calories than the whole-body net energy balance; the SGLT2i group had extra urinary calorie loss, even if the calorie intake was the same as the CON group. The degree of post-prandial EE depends on the proportion of macronutrient intake [35]. Underlining this process, the EE after the protein ingestion was higher compared to the carbohydrate and fat intake [36]. We observed that increased post-prandial EE in the CRIC group associated with a relatively high proportion of protein intake would have an impact on the EE.

### Impact of the route of limited carbohydrate availability (low oral intake or high urinary output) on the respiratory quotient

In the physiological state, the daily whole-body RQ is characterized by diurnal fluctuations, reflective of a metabolically flexible state in which the mitochondria switch the energy substrates (lipids and carbohydrates) based on the nutritional and physiological cues [37]. A high RQ is indicative of glucose oxidation, whereas a low RQ reflects predominant fat oxidation, where the role of amino acids as an oxidative substrate is only minimal. Similar to previous studies [3], the basal RQ was lower in the SGLT2i group compared to the CON, owing to the reliance on enhanced fat oxidation during the fasting state in the SGLT2i group. Although not significant, the basal RQ in the CRIC group tended to be lower compared to the CON group. The basal RQ would be resulted by the whole-body carbohydrate availability, and not the net carbohydrate loss.

Progression toward the postprandial state is accompanied by increased carbohydrate oxidation and a corresponding increase in the RQ in individuals with normal physiology [37]. The differences between the fasting and post-prandial respiratory exchange ratio were similar in SGLT2i and CRIC. This indicates that the amount of remaining carbohydrate in the body was essential for post prandial whole-body energy metabolisms. The difference in the method of energy dissipation, i.e., decreased oral intake or increased urinary glucose loss, did not contribute to the post prandial whole-body energy expenditure.

There were several limitations to the present study. First, we only enrolled in-patients with type 2 diabetes; however, this was necessary because we needed to regularly obtain blood and urine samples to measure the metabolic substrates, EE and RQ at multiple times under controlled conditions. Second, the result of the fasting-to-fed state experiments were obtained from a single meal per day for 12 weeks and may not be generalized to the entire study period. Third, we could not evaluate the effects of ketone bodies or the activities of different glycolytic pathways using tracer methodologies. Finally, the short treatment period (12 weeks) might be insufficient to observe clinically meaningful changes in some variables; therefore, long-term studies are necessary to verify the present results.

In conclusion, we compared the effect of reduced carbohydrate availability using two different approaches (low intake or high urinary output) to assess the insulin requirement to achieve euglycemia, whole-body EE, and metabolic flexibility (RQ) in the fasting and postprandial states in patients with type 2 diabetes. The total daily dose of insulin was higher in the CRIC group compared to the SGLT2i group, possibly due to increased anabolic demands, which related to the high proportion of protein intake compared to the SGLT2i and CON groups. The baseline EE was similar in all groups; however, the postprandial EE increased with SGLT2i and the CRIC diet, which indicates that the proportionally high protein and fat availability altered the substrate preference, rendering it amenable to macronutrient oxidation. Furthermore, the decreased fasting RQ and increased postprandial RQ in both SGLT2i and CRIC groups indicates similar metabolic flexibility in response to similar macronutrient availability.

## Supplementary Information

**Additional file 1:** Study design.

## Data Availability

All study data were registered and available at the National University Hospital Medical Information Network (UMIN Clinical Trials Registry: UMIN0000 27262).
