# Supplementary figures and images for: SGLT2 inhibitor versus carbohydrate-restricted isocaloric diet: reprogramming substrate oxidation in type 2 diabetes
Source: Diabetol Metab Syndr. 2023 Feb 19;15:25. doi: 10.1186/s13098-023-00990-6 (PMC9940379; doi:10.1186/s13098-023-00990-6)

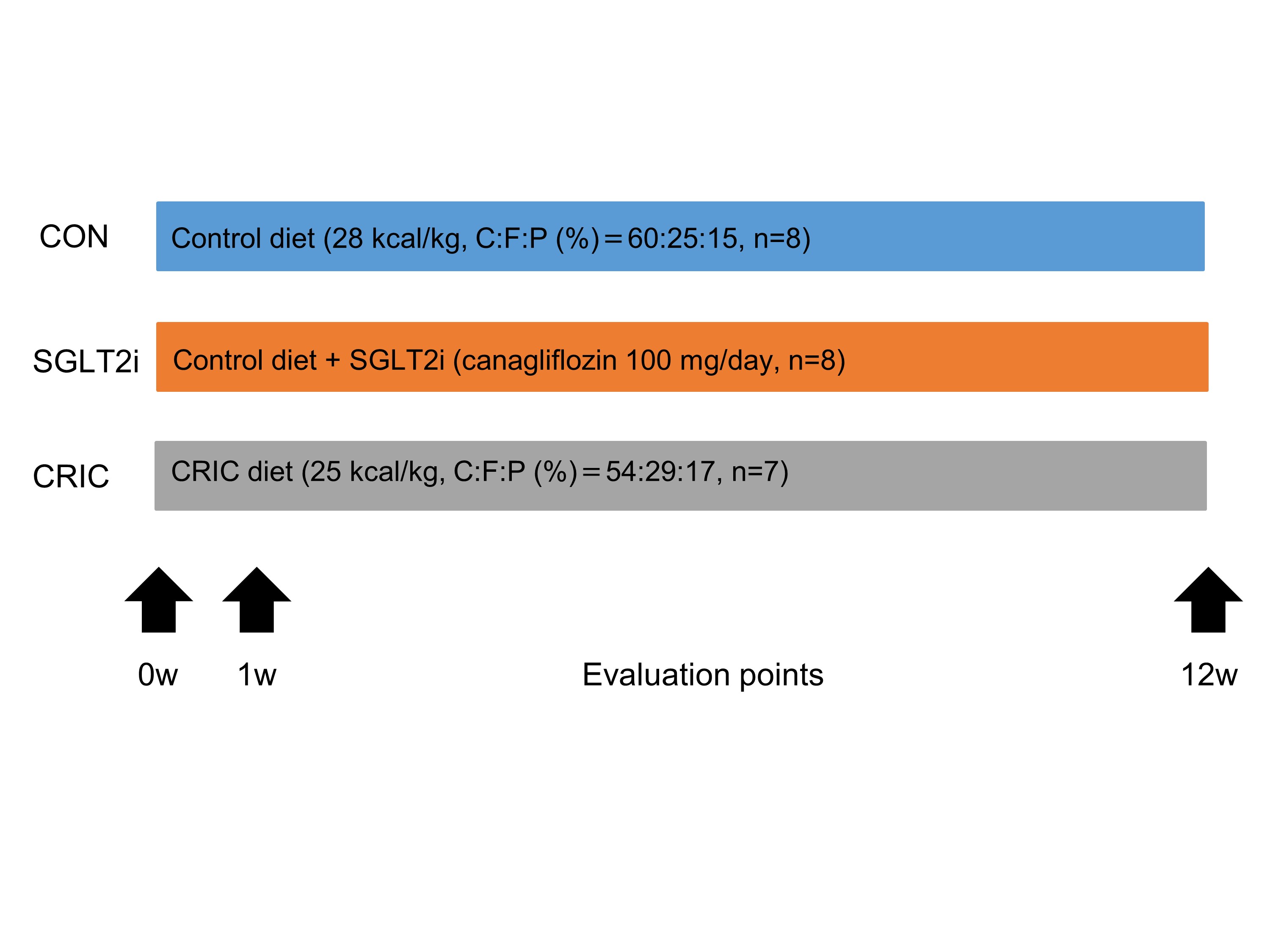

Supplement: Supplementary file 1 — Additional file 1: Study design. [file 13098_2023_990_MOESM1_ESM.jpg]
